# The epidemic characteristics of short stature in school students

**DOI:** 10.1186/s13052-015-0207-6

**Published:** 2015-12-29

**Authors:** Qian Wang, De-yun Liu, Li-qi Yang, Yue Liu, Xian-jun Chen

**Affiliations:** The Second Hospital of Anhui Medical University, Hefei, Anhui 230601 China; The Second Affiliated Hospital of Anhui Medical University, Economical Development Zone, Hefei, Anhui 230601 China

**Keywords:** Short stature, The comparative study, Primary and middle school students, Rural regions

## Abstract

**Background:**

There was no large-scale population survey on the prevalence of short stature in Anhui province yet. To further acquainting the epidemiological character of teenage children short stature in Anhui province, and to provide basis for exploring reasonable intervention measure about children’s height, we took this survey on short stature in primary and middle school students of Anhui province.

**Methods:**

Twelve thousand nine primary and secondary school students in urban and rural aged from 7 to 18 years were recruited. The comparison of short stature in different genders, regions and age groups was done according to genetic metabolic endocrine group of pediatrics branch in Chinese Medical Association (CMA).

**Results:**

The average detection rate of short stature in primary and middle school students was 3.16% in Anhui province. The detection rate was higher in rural area than in urban area, higher in economic backward area than in economic developed areas.

**Conclusion:**

There was no significant difference in detection rate in gender and age.

## Background

Growth and development in children and adolescents is a multi-factor related progress, which is affected by genetic factors and acquired environmental factors. Growth retardation is common disease in pediatrics, with the main performance of short stature. Researches show that growth and development disorder have serious influence on the healthy growth of children and adolescents, and bring harmful effect on the life and work when the sick children grown up, and even become social problem [1–3]. Early discovery of short stature, defining the pathogeny and adopting therapy properly and systemically, not only increase the height of children, but also improve their life quality.

According to the guide on diagnosis and treatment, short stature is defined to individual height that under 2SD of average height or below the third percentile (-1.88SD) of average height in same race, gender and age under similar living environment. Part of them are belong to normal physiological variation [4, 5]. However, large differences exist in the normal value of height in different countries, regions and races. In the global, it’s various in morbidity and pathogenesis of short stature.

Assumed the population base is 1,300,000,000 in China, the population with short stature is 39,000,000 according to the estimation that the prevalence rate is 3 % by the definition of short stature. Actually, it is not the same concept of third percentile and 3 %. It is stated that there is no exact statistical data yet about the overall prevalence of short stature in children and adolescents in our country. The survey about height characteristics in China were carried out sporadically. It’s report that the total detection rate of short stature in - Shanghai was 3.26% in 2003 from a survey about short stature in 6–18 years old children and adolescents [6]. This rate was significantly lower than that published in 2000 by WHO about short stature detection rate in developing country [7], and was lower than the rate of Chile in 2005 [2] and the rate of Wanzhou district of Chongqing in 2014 [8]. However, the rate was higher than that in Zibo district of Shandong province in 2004 [9], and higher than a rate (1 % crowd was in the -2SD scope of average height among same gender and same age) resulting from a survey in 1980s [10]. The prevalence rate of short stature is various among different regions, that some regions show significant differences in urban and rural, boys and girls, while other regions show not. The cause of these differences is related to races, climates and environment. However, it is noted that these differences also highly related to the common standard currently used for short stature in domestic pediatric (that it’s from a survey about 9 cities in 2005). Hence, it is more realistic to formulate the standard of short stature depending on local standard-height.

However, there was no large-scale population survey on the prevalence of short stature in Anhui province yet. To further acquainting the epidemiological character of teenage children short stature in Anhui province, and to provide basis for exploring reasonable intervention measure about children’s height, we took this survey on short stature in primary and middle school students of Anhui province. In this survey, we adopted multistage stratified cluster sampling investigating methods according to the confirmations of sample size, race and location, with the aim to seek the epidemiological characters and differences in different age groups of Anhui students, and to explore and analysis the existing reasons, so to seek the probable reason in Anhui province and provide the basis for the prevention and control of short stature.

In this paper, we screened the short stature in primary and secondary school students of Anhui province according to the standard for short stature established by genetic metabolic endocrine group of pediatrics branch in Chinese Medical Association (CMA). The survey on distribution characteristics of short stature in Anhui students was carried out in different regions, genders and age groups.

## Methods

This work was supported by the Ethic committee of The Second affiliated Hospital of Anhui Medical University.

By the stratified random cluster sampling method, total 12,009 students from urban and rural primary and middle school were chosen from Suzhou city in the north, Hefei city in the middle and Dongzhi county in the south of Anhui province. Among them, aged 7–12 years old as pupil group, aged 13–15 years old as junior middle school students group, and aged 16–18 years old as senior middle school students group. All of the students were divided into 4 group of classes according to rural and urban, male and female. Equality sampling was taken from three regions-economic developed region-Hefei city, economic averaged region-Suzhou city, and economic undeveloped region-Dongzhi county. Subjects were consisted of 3008 city boys, 2887 city girls, 3088 country boys, and 3026 country girls, without limb disabilities. The excluding criteria contained limb disabilities and people unwilling to attend this survey.

The survey crew were trained under unified standard before taking measurement. Averaging three times measurements for height, the data were accurate to 0.1 cm. When measuring the height, the subject was standing on the baseboard of height meter, with the posture up-right and two eyes straight ahead, and with the heel, sacral region and the middle between shoulder blades leaning on the stand column of height meter. Surveyor stood by one side of subject, whose head was fit to the position that the upper edge of tragus was parallel with the lowest point of the lower edge of eyes, then moved the level board of height meter to the top of subject’s head, and made the level board in proper tightness. So subject’s height was measured exactly. According to the standard of short stature established by genetic metabolic endocrine group of pediatrics branch in CMA, meeting one of the following conditions was diagnosed to short stature: 1, height is under 2SD of average height in same race, gender and age; 2, height is below the third percentile of average height in same race, gender and age. The short stature groups were screened out in various region, age and gender groups, other samples were incorporated into normal group.

### Statistic analysis

The data were analyzed by SPSS 16.0 software. We took multi-factor logistic analysis on the different demographic variables of short stature in primary and secondary school students of Anhui province.

## Result

### Overall epidemic status of short stature in primary and secondary school students of Anhui province

There were 380 subjects who were detected to short stature in primary and secondary school students of Anhui province. The detection rate was 3.16%, with the feature that it’s highest in Dongzhi county, lowest in Suzhou, rural was higher than urban, there was no difference between male and female, neither between different age groups. As shown in Table 1.

**Table 1:** Multi-factor logistic analysis on the different demographic variables of short stature in primary and secondary school students of Anhui province

Demographic variables	Short stature			
	n (%)	OR	(95 %CI)	P
Total	380 (3.16)			
Region				
Hefei	94 (2.62)	0.565	0.440–0.725	0.000
Suzhou	83 (2.14)	0.465	0.359–0.603	0.000
Dongzhi	203 (4.47)	1.00		
Urban and Rural				
Urban	151 (2.56)	0.675	0.547–0.832	0.000
Rural	229 (3.75)	1.00		
Gender				
Male	186 (3.05)	0.913	0.744–1.121	0.386
Female	194 (3.28)	1.00		
Age groups				
7–9 years old	92 (2.95)	0826	0.618–1.103	0.195
10–12 years old	91 (2.95)	0.848	0.635–1.131	0.262
13–15 years old	97 (3.20)	0.910	0.685–1.211	0.519
16–18 years old	100 (3.60)	1.00		

*Note*: all compared to normal group

### The detection rates of short stature in different economic development level regions

The total detection rate of short stature showed: Dongzhi > Hefei > Suzhou, no matter in boys or girls. The rate was highest in Dongzhi. Please see Table 2.

**Table 2:** The detection rate of short stature (%) male and female students in three regions of Anhui province

Region	Gender	Detection rate
		Short stature
Hefei	Male	2.60 (47/1811)
	Female	2.64 (47/1782)
	Total	2.62 (94/3593)
Suzhou	Male	1.97 (39/1984)
	Female	2.33 (44/1890)
	Total	2.14 (83/3874)
Dongzhi	Male	4.35 (100/2301)
	Female	4.60 (103/2241)
	Total	4.47 (203/4542)

**Fig. 1:**
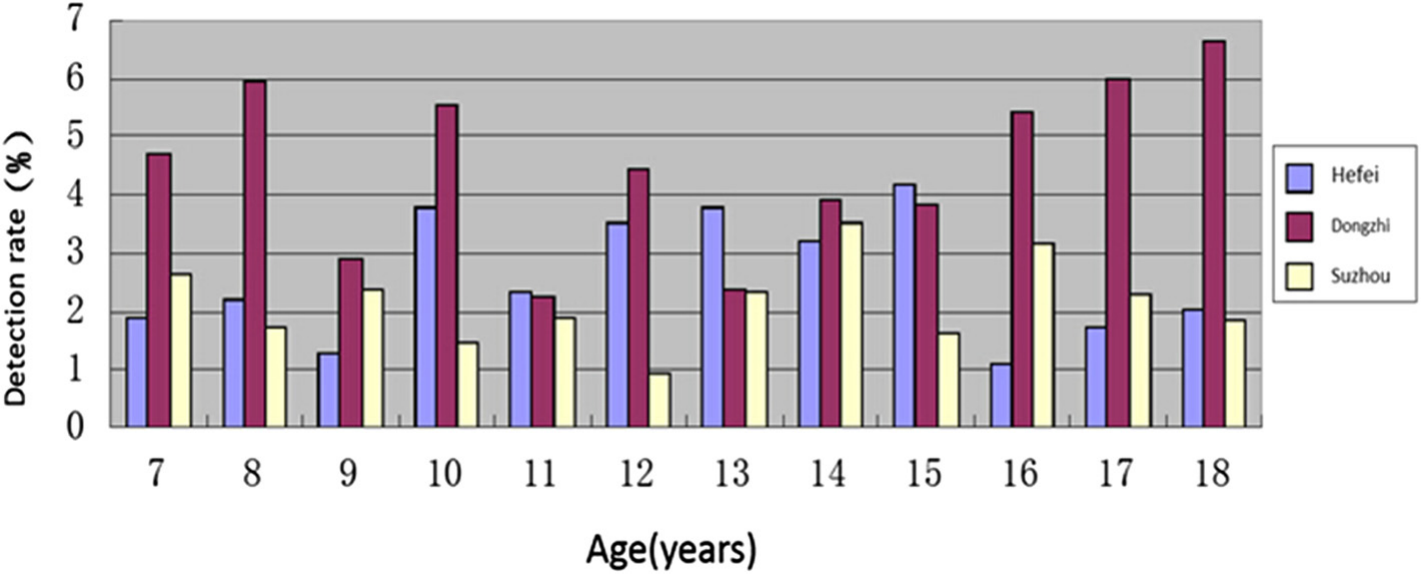
The detection rate of short stature (%) in primary and secondary students from three regions of Anhui province

As shown in Fig. 1, the detection rate of short stature in Hefei showed the trend of high in middle and low on both ends, while Dongzhi showed the trend of low in middle and high on both ends. There was no significant correlation between the detection rate of short stature and age distribution in Suzhou. The rates were higher in pupil group (7–12 years age) and senior middle school group (16–18 years age) of Dongzhi than in Hefei and Suzhou. There were no differences among three regions in junior middle school group (13–15 years age).

### The detection rates of short stature in urban and rural

As shown in Table 3, the rate of short stature in aged 7–18 years primary and secondary students of Anhui province in 2014 were: 2.53 % in city boys, 2.60 % in city girls, 3.56 % in country boys and 3.93 % in country girls. The detection rates of short stature showed: country girls> country boys> city girls> city boys. The detection rate of short stature showed higher in rural than in urban no matter boys or girls. Among them, the rate of country boys was higher than city boys’ for 1.03 %; the rate of country girls was higher than city girls’ for 1.33 %.

**Table 3:** The detection rate of short stature (%) in male and female primary and secondary students of Anhui province urban and rural in 2014

Urban and Rural	Gender	Detection rate
		Short stature
Urban	Male	2.53 (76/3008)
	Female	2.60 (75/2887)
	Total	2.56 (151/5895)
Rural	Male	3.56 (110/3088)
	Female	3.93 (119/3026)
	Total	3.75 (229/6114)

**Fig. 2:**
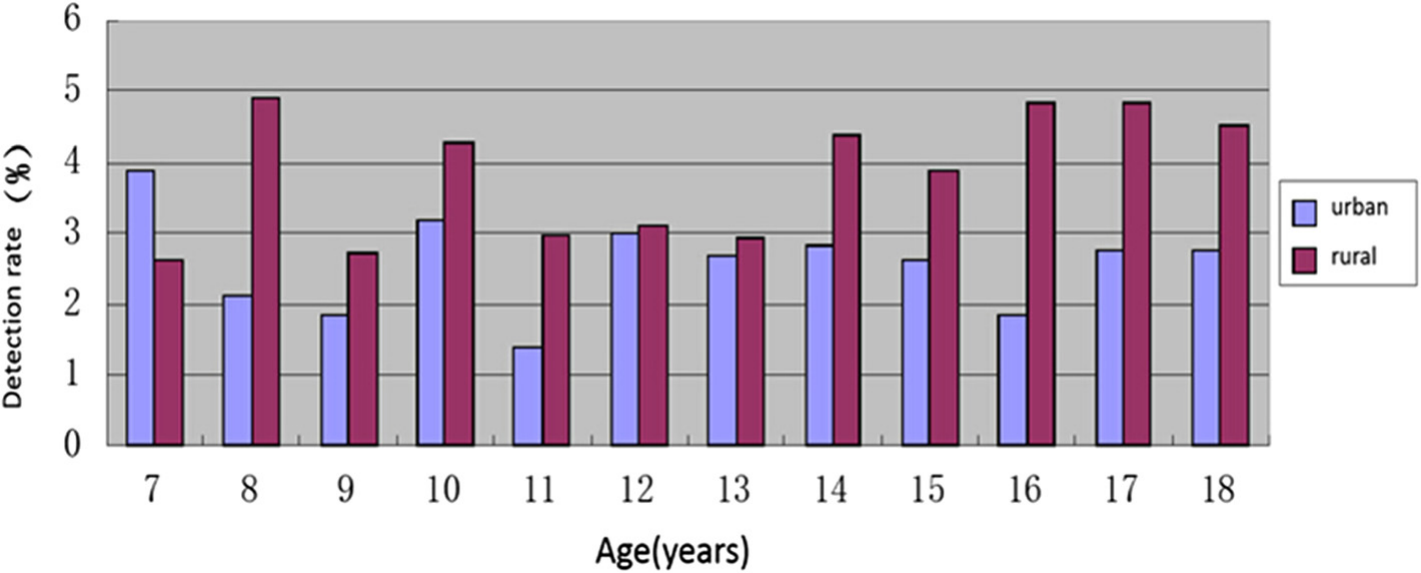
The detection rate of short stature in primary and middle school students of Anhui province

As shown in Fig. 2, except 7-years age group, the detection rate of short stature was higher in rural than in urban in other age groups of primary and middle school students of Anhui province.

### The detection rates of short stature in different age groups

As shown in Table 4, in male subjects, no matter in urban or in rural, the highest rate was 13–15 age group (junior middle school students); the lowest group in urban was 16–18 age group (senior middle school students); the lowest group in rural was 7–9 age group (primary school students). In female subjects, the detection rate of short stature in urban was mainly focus on primary school stage, the highest rate emerged in 7–9 age group, however, the highest rate in rural was detected in senior middle school students.

**Table 4:** The detection rate of short stature in boys and girls of different age groups in Anhui province urban and rural

Urban and rural	Age group (year)	Boys	Girls
Urban	7–9	2.22 (17/766)	3.08 (23/746)
	10–12	2.41 (19/790)	2.65 (20/755)
	13–15	3.26 (25/766)	2.05 (15/730)
	16–18	2.19 (15/686)	2.59 (17/656)
Rural	7–9	2.96 (23/778)	3.49 (29/832)
	10–12	3.35 (26/777)	3.43 (26/758)
	13–15	4.22 (33/782)	3.19 (24/752)
	16–18	3.73 (28/751)	5.85 (40/684)

**Fig. 3:**
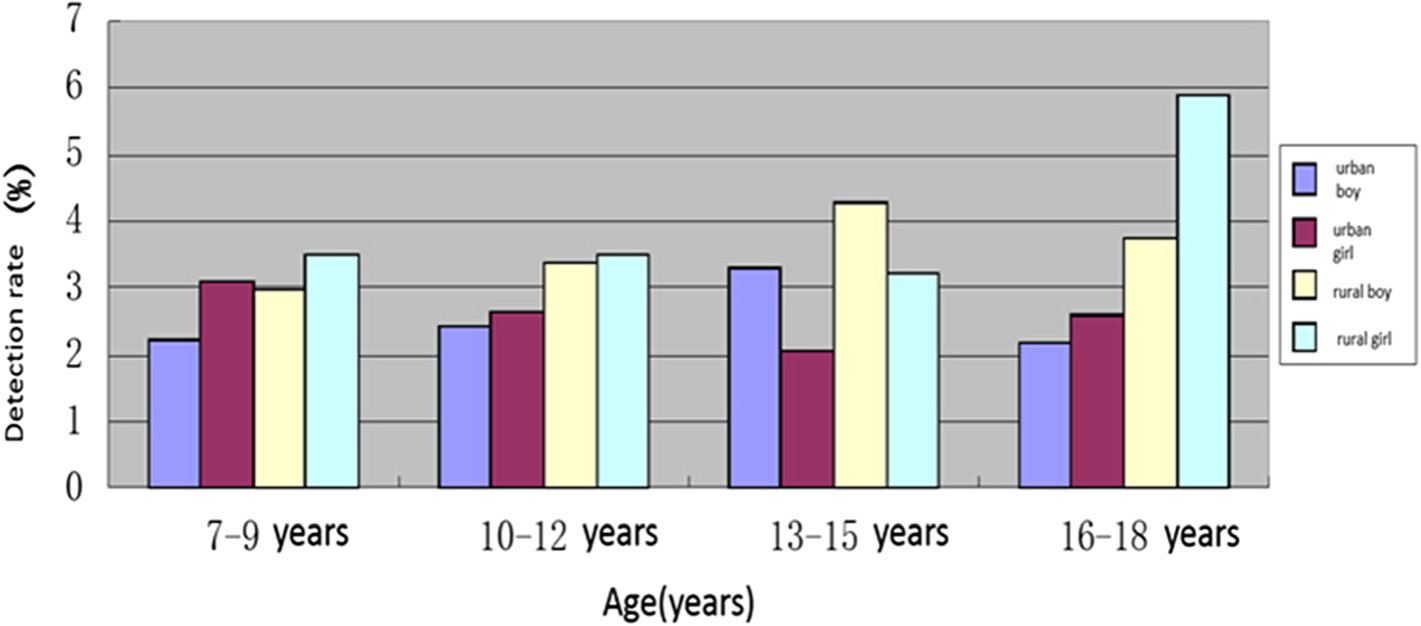
The detection rate of short stature in primary and middle school students of different age groups in Anhui province

Rural students in every age group were in the top list of short stature occurrence. Among them, the rate of rural female senior high school students was up to 5.85 %. The rates in 7–12 age group in urban and rural were similar in boys and girls. In senior high school students, there was no difference in short stature rate between boys and girls in urban, however, it’s significantly higher in girls than in boys in rural. Please see Fig. 3.

## Discussion

The causes of short stature are complex, containing genetic factor (race, parents, etc.), environmental factor (region, food, living habit, climate, etc.), nutritional factors (intrauterine growth restriction, postnatal malnutrition), idiopathic short stature and disease factors (endocrine abnormalities, chromosomal disorders, inherited metabolic diseases, skeletal dysplasia, chronic systemic disease, etc.) [4, 11]. Short stature will seriously affect the healthy growth of children and adolescents that it will damage the linear growth of height, leading to physical and mental obstacle and social deficit [11]. Early detection of short stature and defining the pathogens will provide the basis for the prevention and control of the disease and formulating relevant public health policy.

Research showed that there was no significant difference between boys and girls, but there was significant differences between urban and rural in the detection rate of short stature, which was higher in rural. It might own to the low growth and development level in rural students that inheriting from parents, dietary structure, nutriture and environmental factors which influence the growth and development of rural students.

This survey was sampled equally from economic developed region-Hefei city, economic averaged region-Suzhou city, and economic undeveloped region-Dongzhi county. The detection rates of short stature were Dongzhi> Hefei> Suzhou. Dongzhi county had the highest rate, which was conform to the economic level. However, the rate of Hefei was higher than Suzhou. Besides sampling error in survey, we think that economy may not the critical factor in the determent of stature, which is also influenced by race, living habit and environment, all of the above factors impact the forward or postpone arrival of puberty [12], which has direct effect on height changing. The complicated influence factors may bring the various appearance of short stature in Hefei and Suzhou.

This survey about the prevalence rate of short stature in primary and middle school students in Anhui province (3.16 % in total, 2.53 % in city boys, 2.60 % in city girls, 3.56 % in country boys and 3.93 % in country girls) is significantly lower than that published in 2000 by WHO about short stature detection rate in developing country (32.5 %) [7], and is lower than the rate of Chile in 2005 (12 % in boys, 20.4 % in girls) [2] and the rate of Wangzhou district of Chongqing in 2014 (23.46 % in total, 16.74 % in urban and 33.66 % in rural of 70,952 primary and middle school students in 5–18 age) [8]. However, the rate is higher than that in Zibo district of Shandong province in 2004 (0.64 % in total, 0.8 % in urban and 0.41 % in suburb of 15,479 subjects) [9] and higher than a rate (1 % crowd was in the -2SD scope of average height among same gender and same age) resulting from a survey in 1980s [10]. Meanwhile, the result of a survey on 6–18 years age children and adolescents of Shanghai in 2003 (3.26 % in total, 2.57 % in urban and 3.75 % in suburb) is similar to ours. The prevalence of short stature is various in different regions. Some regions have differences in urban and rural, male and female, however, others are not. This survey showed significant difference in urban and rural, and no significant difference in male and female about the prevalence of short stature in Anhui province. These differences may relate to economic level and race in different regions. Further research is needed.

Data from the Education Department of Anhui province show that, there are 10,420,000 primary and middle school students in Anhui, containing 5,840,000 pupils, 4,580,000 middle school students. According to the prevalence rate of 3.16 %, there are about 329, 272 students with short stature in primary and middle school students, that 184, 544 in primary school students and 44,728 in middle school students. The huge number causes worry about the severe condition of short stature in Anhui province. There are differences in different regions, urban and rural, but no significant difference in different gender and ages. Hence, Anhui provincial government agencies need to formulate comprehensive prevention plan to short stature that carry out the measures such as health education, reasonable diet, fitting exercise, adequate sleep and improving life style. Conditions permit, further examination in hospital is suggested, for exploring and analyzing the probable etiology and risk factors, providing the basis for establishing relevant public health policy, and guiding medical staff adopting suitable and effective treatment for short stature, so to save medical resource.

## Conclusion

Based on above information, there are large differences in the morbidity of short stature around the world, even among cities in one country. Analyzing reasons for the diversity, besides race, economy, and environment, it’s closely related to the common standard currently used for short stature in domestic pediatric (that it’s from a survey about 9 cities in 2005). Therefore, it is more realistic to establish the standard for short stature according to regional height standard. Establishing Anhui local standard on the basis of the national standard is beneficial to the screening of short stature in Anhui province, and provide more scientific basis for the prevention and etiological analysis of short stature.

However, in this research, other factors such as race, living habit, natural environment and climate, etc. were not taken into account. Next step, we will consider multifaceted element so to limit the incomplete analysis of the reason to short stature in this survey.
